# Authorship in Oral and Maxillofacial Surgery

**DOI:** 10.1007/s12663-021-01538-9

**Published:** 2021-03-16

**Authors:** Pravesh S. Gadjradj, Mamta Jalimsing, Sandhia Jalimsing, Istifari Voigt

**Affiliations:** 1 Department of Neurosurgery, Leiden University Medical Center, University Neurosurgical Center Holland (UNCH), Albinusdreef 2, 2333 ZA Leiden, The Netherlands; 2 Faculty of Medicine, Erasmus MC: University Medical Center Rotterdam, Rotterdam, The Netherlands

**Keywords:** Authorship, Guidelines, Oral and maxillofacial surgery

## Abstract

**Background and Objective:**

According to the International Committee of Medical Journal Editors (ICMJE), authorship should be offered based on fulfilling four criteria. Honorary authorship (HA) is a term used for authors enlisted who did not fulfill these criteria. The objective of this study was to determine the proportion of HA in the field of oral and maxillofacial surgery.

**Material and Methods:**

In 2020, a twenty-two question survey was sent to corresponding authors of four high-impact journals in the field of oral and maxillofacial surgery. The survey covered (1) demographics, (2) awareness of authorship guidelines and decision-making of authorship, and (3) honorary authorship.

**Results:**

The response rate was 24.8%. Of the respondents, 81.1% was aware of the issue of guidelines on authorship, while 56.3% was aware of the issue of HA. Yet, 15.5% of the respondents felt that one or more of their co-authors did not deserve authorship based on the ICMJE-guidelines.

**Conclusion:**

Based on the estimated proportions of HA, attempts should be made by universities, medical journals and individual researchers to further reduce authorship misuse.

## Introduction

Authoring scientific publications can provide clinicians opportunities to further their clinical or scientific career. According to the International Committee of Medical Journal Editors (ICMJE), authorship should be offered based on fulfilling four criteria [1]:

1. “1. Substantial contributions to the conception or design of the work; or the acquisition, analysis, or interpretation of data for the work; AND
2. Drafting the work or revising it critically for important intellectual content; AND
3. Final approval of the version to be published; AND
4. Agreement to be accountable for all aspects of the work in ensuring that questions related to the accuracy or integrity of any part of the work are appropriately investigated and resolved [1].”

Honorary authorship (HA) is a term used for authors enlisted who do not fulfill these criteria. As HA gives inappropriate credit to authors, it is classified as scientific misconduct in the medical literature [2]. It is unknown to what extent HA is an issue in the maxillofacial literature.

Therefore, the aim of the current study is to analyze the proportion of HA in different journals in various journals in the oral and maxillofacial surgery.

## Material and Methods

In 2020, a twenty-two question survey was sent to corresponding authors of articles published in 2019 in four high-impact journals in the field of oral and maxillofacial surgery. Editorials, manuscript correspondence and articles with only one author were excluded. The survey covered (1) demographics, (2) awareness of authorship guidelines and decision-making of authorship, and (3) honorary authorship [3–6]. The survey contained a list of “non-authorship” tasks such as obtaining funding. Authors performing one or more of these tasks and not contributing to the manuscript otherwise, are defined as “ICMJE-defined HA.” Furthermore, respondents were asked if they felt that one or more of their co-authors did not deserve authorship. This was defined as “self-perceived” HA.

## Results

### Demographics

**Fig. 1:**
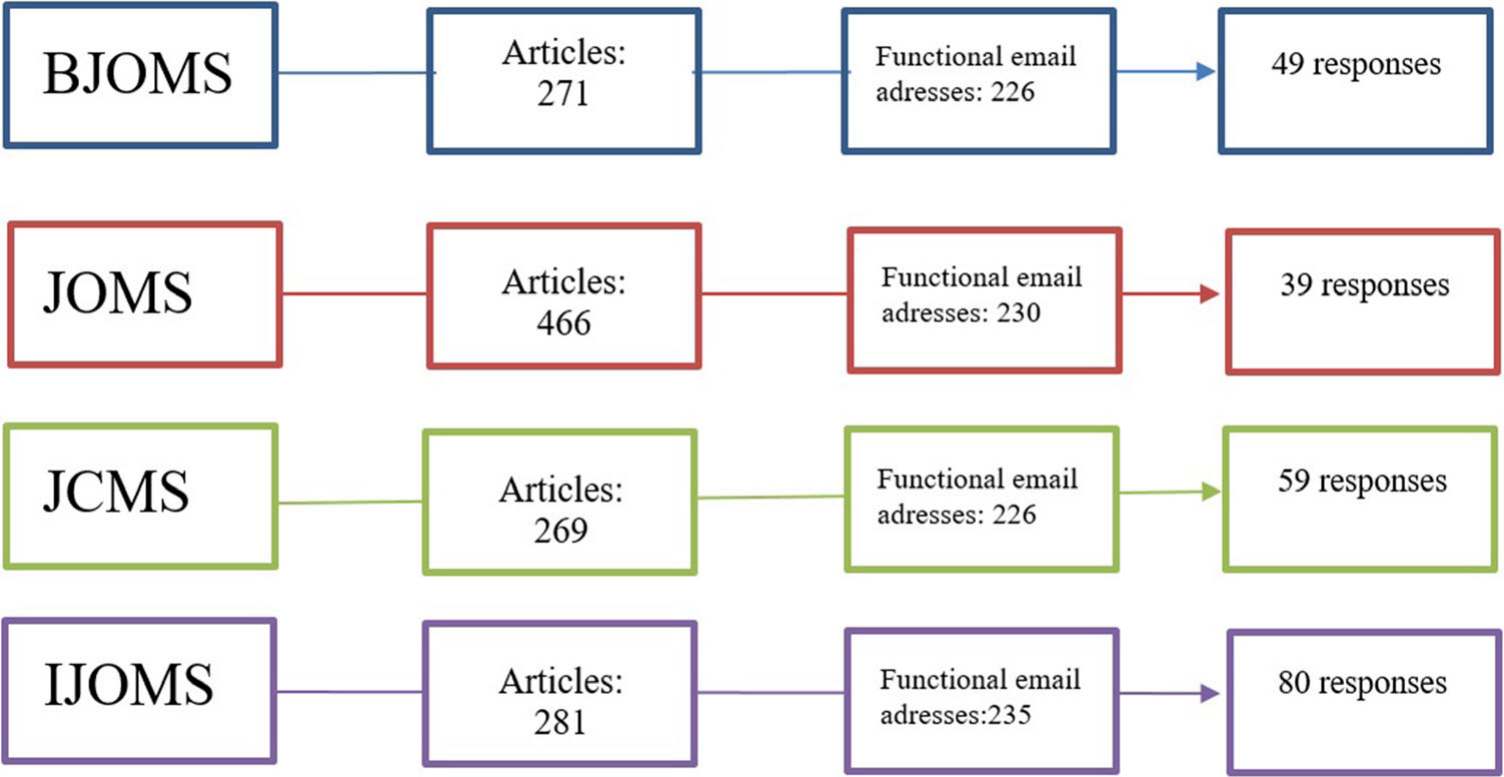
Flowchart of the study procedures. *BJOMS* British Journal of Oral and Maxillofacial Surgery, *JOMS* Journal of Oral and Maxillofacial Surgery, *JCMS* Journal of Cranio-Maxillofacial Surgery, *IJOMS* International Journal of Oral and Maxillofacial Surgery

In total, 227 out of the 914 sent surveys were answered, leading to a response rate of 24.8% (see Fig. 1). Most respondents were employed as oral and maxillofacial surgeon (65.2%), while they represent working locations from 40 different countries (see Table 1).

**Table 1:** Answers on questions regarding demographics, authorship guidelines and authorship decision-making

Question	*N* (%)
*Peer reviewed articles authored*	227
< 5	35 (15.4%)
6 to 15	63 (27.8%)
16 to 25	29 (12.8%)
> 25	100 (44.1%)
*Primary profession*	227
Oral and Maxillofacial surgeon	148 (65.2%)
Dentist	23 (10.1%)
Researcher	26 (11.5%)
Other	3 (1.3%)
*Tenure (years)*	227
1 to 2	18 (7.9%)
3 to 5	35 (15.4%)
6 to 10	41 (18.1%)
> 10	133 (58.6%)
*Aware of the ICMJE-guidelines on authorship*	227
Yes	184 (81.1%)
No	43 (18.9%)
*If unaware, aware of other authorship guidelines*	104
Your institution guidelines	74 (71.2%)
No guidelines are followed	21 (20.2%)
Other	9 (8.7%)
*Before taking the survey, aware of the general issue of honorary authorship*	192
Yes	108 (56.3%)
No	84 (43.8%)
*Is there a senior member, who is automatically enlisted as author on all manuscripts?*	226
Yes	56 (24.8%)
No	166 (73.5%)
Don’t Know	4 (1.8%)
*If so, do you feel this is justified?*	157
Never justified	47 (29.9%)
Rarely justified	33 (21.0%)
Sometimes justified	43 (27.4%)
Most of the time justified	18 (11.5%)
Always justified	16 (10.2%)
*Ever been involved in authorship dispute*	227
Yes	64 (28.2%)
No	162 (71.4%)
Other	1 (0.4%)
*Has a professional relationship been damaged because of an authorship dispute?*	222
Yes	164 (73.9)
No	58 (26.1%)
*Regarding your paper, who decided the order of authorship?*	227
First author	66 (29.1%)
Senior author	51 (22.5%)
Authors decided as a group	91 (40.1%)
The funding source of this study	4 (1.8%)
Other	15 (6.6%)
*What was your primary role in the article?*	227
Wrote all or most of the article	161 (70.9%)
Wrote minor parts of the article	3 (1.3%)
Only revised the article and made corrections and changes in content	9 (4.0%)
I supervised the writing of others	19 (8.4%)
Performed majority of data collection/ analysis	14 (6.2%)
Other	21 (9.3%)
*Gender*	227
Male	170 (74.9%)
Female	57 (25.1%)
*Continent employed*	227
Africa	8 (3.5%)
Asia and Oceania	64 (28.2%)
Europe	100 (44.1%)
North America	20 (8.8%)
South America	35 (15.4%)
*Study funding (multiple answers possible)*	
(Pharmaceutical) Industry	0
University sponsored	48 (21.1%)
No funds obtained	172 (75.8%)
Other	12 (5.3%)
*What criteria did you use to decide the order of authorship? The authors are listed*	226
In the order of the amount each contributed	97 (42.9%)
In the order of the amount each contributed, except the last author, who is the most senior in the group but did not contribute to the study	15 (6.6%)
In the order of the amount each contributed, except the last author, who provided the concept, supervision and responsibility for all steps	109 (48.2%)
In alphabetical order	1 (0.4%)
Other	4 (1.8%)
*Did anyone suggest to include an honorary author?*	224
Yes	39 (17.4%)
No	185 (82.6%)
*Did any of your coauthors performed only one or more “non-authorship” tasks and nothing else related to study design, manuscript preparation etc.?*	227
BJOMS	22 (44.9%)
JOMS	16 (41.0%)
JCMS	34 (57.6%)
IJOMS	41 (51.3%)
*Which tasks were performed? (multiple answers possible)*	
Supervising/ recruiting coauthors	28 (12.3%)
Obtaining funding or material support	15 (6.6%)
Recruiting study subjects	34 (15.0%)
Performing cases used in the study	44 (19.4%)
Contributing illustrations	23 (10.1%)
Reviewing the manuscript	78 (34.4%)
Approving the manuscript before submission	57 (25.1%)
Signing statement of copyright transfer	35 (15.4%)
*Do you believe that any of your coauthors enlisted for the current article did not make sufficient contributions to merit coauthorship?*	226
BJOMS	8 (16.3%)
JOMS	2 (5.5%)
JCMS	8 (13.6%)
IJOMS	17 (21.3%)
*Selection of answers on “what does authorship mean to you?”*	
“That the authors contribute NO freeloaders!”	
“It means a lot, especially to be first author on a publication. This is, as specific criteria in terms of publications are requested by the university. It is also important in which journal the paper is published. Higher ranked journals bring more points with regards to the university criteria than lower ranked journals.”	
“I have previously felt pressure to put senior department members as authors on papers for which they did not contribute. This practice should and must change.”	
“It provides me a sense of accomplishment and respect.”	
“My work my name. Not my work, don't want my name anywhere!”	

### Awareness of Authorship Guidelines and Decision-Making of Authorship

Before the survey, 81.1% was aware of the ICMJE-guidelines, while 56.3% was aware of the issue of HA. Regarding the publication surveyed, the order of authorship was mostly decided by authors as a group (40.1%), followed by the first author (29.1%) and senior author (22.5%) deciding. The order of authors was mostly determined by the amount each contributed (42.9%).

### Honorary Authorship

Overall, the proportion of self-perceived HA was 15.5%, which ranges from 5.5 to 21.3% among the journals surveyed, while the proportion ICMJE-defined HA was 49.8% ranging from 41.0% to 57.6%. Continent of employment and the journal surveyed were not associated with HA.

**Fig. 2:**
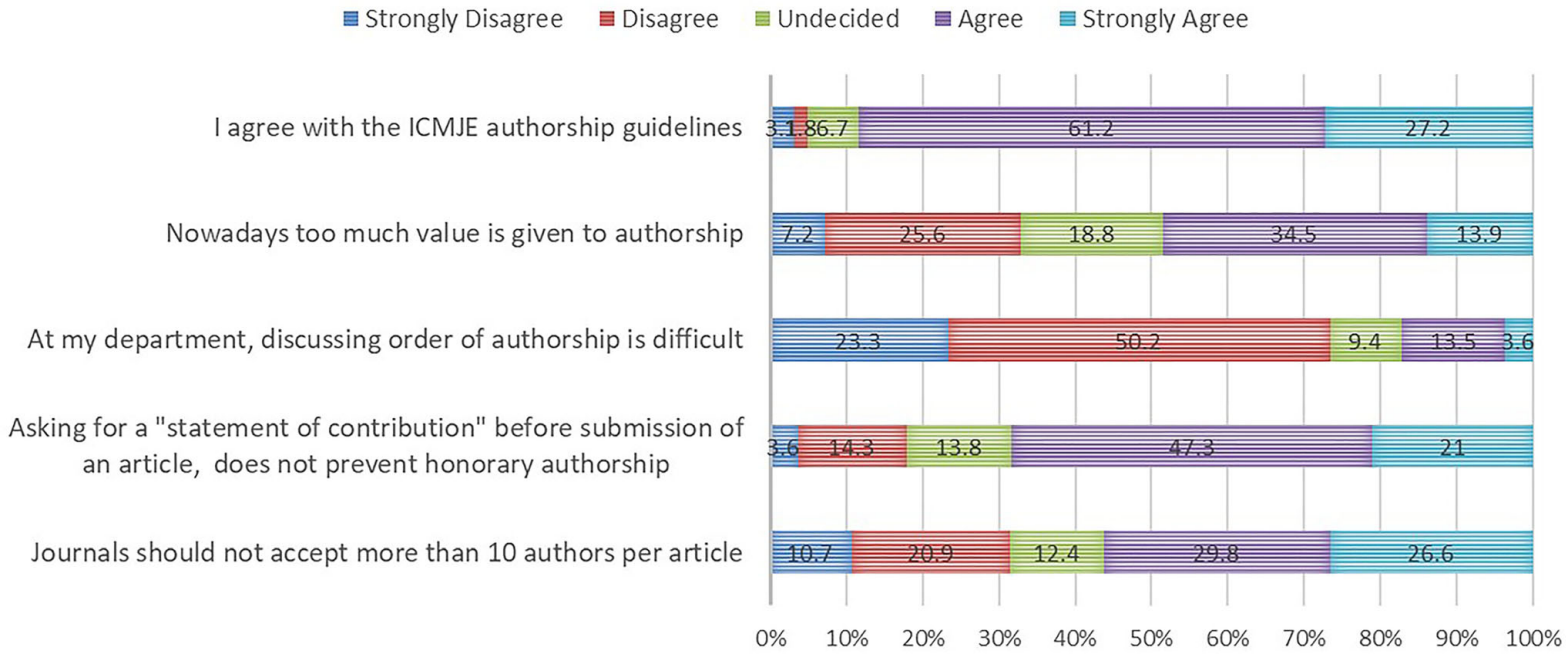
Opinions on authorship issues

Figure 2 gives an overview of opinions on authorship issues. Most respondents (strongly) agreed (68.3%) that journals asking for “a statement of contribution” before submitting a work, does not prevent HA.

## Discussion

The present study shows that the vast majority of the respondents are aware of the ICMJE-guidelines and agree with them. Despite this awareness of authorship guidelines, the proportion of self-perceived HA was 15.5%, while the proportion of ICMJE-defined HA was 49.8%.

Some limitations have to be acknowledged. First, the response rate is 24.8% which may introduce selection bias. Second, we surveyed corresponding authors. Corresponding authors might consist of more senior authors which can give a lower estimate of HA. Finally, recall bias could be introduced due to the retrospective nature of the survey. Previous published studies suggest some solutions to reduce the proportion of HA. For example, a solution might be the implementation of courses on publication ethics for researchers. Another solution might be the referral to and endorsement of authorship guidelines by medical journals. Furthermore, implementing a support system to discuss and resolve authorship disputes may also help reduce the proportion of HA [7].

Based on the estimated proportions of HA, attempts should be made by universities, medical journals and individual researchers to further reduce authorship misuse. These attempts should not only focus on raising awareness of authorship guidelines but also on facilitating open discussions of authorship issues for both junior and senior researchers.

## Declarations

## Conflict of interest

The authors have no conflicts of interest.

## Abbreviations

HA: Honorary authorship
ICMJE: International Committee of Medical Journal Editors
BJOMS: British Journal of Oral and Maxillofacial Surgery
JOMS: Journal of Oral and Maxillofacial Surgery
JCMS: Journal of Cranio-Maxillofacial Surgery
IJOMS: International Journal of Oral and Maxillofacial Surgery
